# Hypoplastic coronary artery disease, as a cause of sudden death

**DOI:** 10.4322/acr.2023.440

**Published:** 2023-07-19

**Authors:** Moirangthem Sangita, Jayanthi Yadav, Jai Kumar Chaurasia, Arneet Arora, Afsar Jahan, Mrinal Patnaik

**Affiliations:** 1 All India Institute Medical Sciences, Bhopal, Forensic Medicine and Toxicology, Bhopal, India; 2 All India Institute Medical Sciences, Bhopal, Pathology and Lab Medicine, Bhopal, India

**Keywords:** Coronary Disease, Death, Sudden, Autopsy, Pathology

## Abstract

Hypoplastic coronary artery disease (HCAD) is a rare coronary artery anomaly that may be the cause of sudden death. It can involve a single or all coronary arteries. This anomaly may cause circulatory insufficiency leading to myocardial infarction. HCAD has no symptoms or may exhibit cardiovascular signs like syncope, dyspnea, chest discomfort, or dizziness. It is often diagnosed at autopsy, and early diagnosis is made with a coronary angiogram. We report HCAD as the cause of the sudden death of a 25-year-old female with a history of loss of consciousness following exertion. On autopsy, all the coronary arteries’ lumen was narrowed with thin vessel walls. Histopathological examination shows an underdeveloped and missing muscular layer of the left anterior descending and circumflex coronary arteries’ vascular wall. Many cases of HCAD diagnosed by radiographic imaging in living patients have been reported in the literature, but a structural anomaly of coronaries leading to HCAD has not been reported. We report a case of HCAD describing the histopathological examination findings of the vascular wall of coronary vessels illustrating the structural difference.

## INTRODUCTION

Cardiac diseases represent 45-50% of all sudden deaths.^1^ A small percentage of the overall population (1%) is affected by congenital coronary artery abnormalities.^2-5^ Congenital coronary artery abnormalities (CAA) are a broad category of malformations present at birth and range in severity and clinical symptoms.^6^ Congenital abnormalities include anomalous origin, absence of coronary artery, fistula, myocardial bridging, aneurysm, and hypoplastic coronary artery disease.^2,4^ These anomalies may pose the myocardium at a higher risk of ischemia and sudden death in the young population and athletes. Angelini^7^ classified coronary artery anomalies into three categories according to their anatomy: i) anomalies of origin and course, ii) anomalies of intrinsic coronary artery anatomy, and iii) anomalies of coronary termination. About 2.2% of people with coronary abnormalities have been documented to have hypoplastic coronary artery disease.^8^

Coronary hypoplasia, also known as hypoplastic coronary artery disease (HCAD), is a congenital aberration of the intrinsic anatomy of these arteries and is characterized by the presence of a single or more than one abnormally tiny or underdeveloped coronary arteries.^9^ The typical coronary luminal diameter is 1.5 mm. However, this measure may vary depending on the dominance of the coronary arteries and the afflicted artery or arterial segment.^8,10,11^ Hypoplastic coronary arteries may also occur in association with other abnormalities. The severity of the disorder and the number of involved coronary arteries will reflect the clinical signs. The illness may be symptomless or exhibit cardiovascular symptoms like syncope, dyspnea, chest discomfort, or dizziness.

Early diagnosis can be made with a coronary angiogram. Both clinicians and pathologists should seek for this disease to find out any probable coronary reasons for sudden death and arrhythmia, which may have been unnoticed. This entity is occasionally diagnosed at the autopsy and may even, in this setting, be a misdiagnosis. Diagnosing such an anomaly by gross examination is difficult unless an accurate measurement or evidence of a scar in the form of an old infarct is seen. However, the histopathological examination (HPE) of the coronary vessels and the surrounding tissues can detect the anomaly and establish the diagnosis.

We searched on PubMed and Google Scholar using the keywords “histopathological examination of hypoplastic coronary artery,” and “hypoplastic coronary artery” and found 147 articles. However, only 20 articles were related to coronary hypoplasia, and in these, the diagnosis was reached by different diagnostic modalities. Only two articles described the microscopic findings of myocardial infarction,^4,5^ but none described the microscopic findings of the wall of the hypoplastic coronary artery.

## CASE REPORT

This is a case of a 25-year-old, obese female, 148 cm in height and weighing 87 Kg (BMI: 39.7), who arrived dead at the hospital. She was found unresponsive, lying on the floor while cleaning the house. A postmortem examination was performed to find the cause of death. She had a history of fainting spells and exertional dyspnea, but a medical evaluation was never done.

### Autopsy Findings

**Figure 1:**
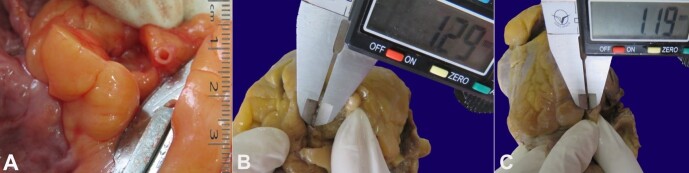
Gross view and measure of the coronary arteries after fixation (B, C, and D). **A -** thinned out proximal left anterior descending artery of the fresh heart during the autopsy; **B -** left anterior descending artery measuring the diameter of 1.29 mm; **C -** left circumflex artery measuring the diameter of 1.19 mm.

On postmortem examination, the female body was short and heavily built. Her face and neck were plethoric. The brain, lungs, liver, kidneys, and spleen were congested. Heart weight was 291g (normal range 192g-424g).^12^ All coronaries were thinned out, with narrowed luminal diameters (Figure 1A). The left anterior descending artery’s diameter was 1.29 mm (Figure 1B), the left circumflex artery was 1.19 mm (Figure 1C), right coronary artery was 1.02 mm when measured 1 cm away from their respective origins. The coronary ostia are normal in position; the diameter of the right coronary ostia was 2.41 mm in diameter (normal range 3.17-3.9 mm),^13^ and the left coronary ostia was 1.89 mm (normal range 4.1mm-4.96mm)^13.^ The left ventricular wall thickness was 13.36 mm, and the right ventricle wall of 6.38 mm. The whole heart and tissues from lungs, kidneys, and liver were kept for histopathological examination.

**Figure 2:**
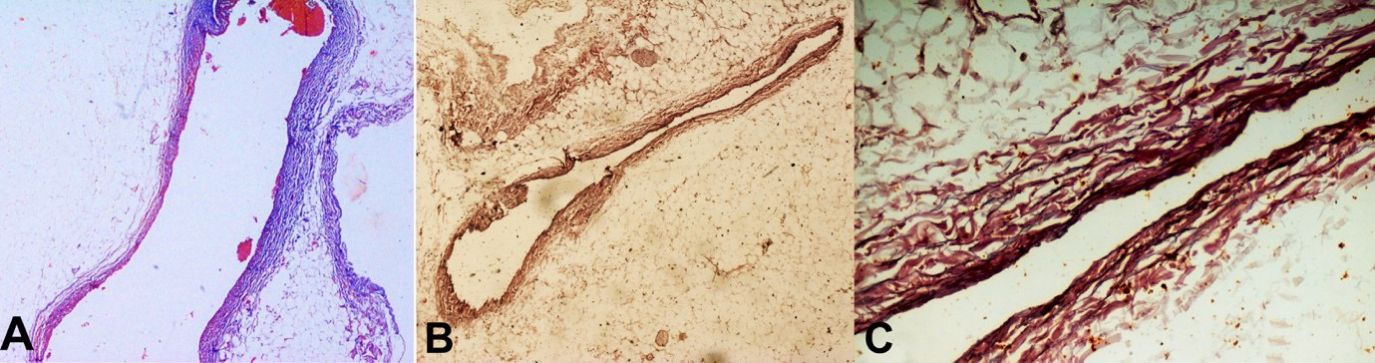
Photomicrographs of the coronary arteries **A**, **B**, and **C -** shows the left anterior descending artery with thin tunica media (**A -** Masson Trichrome stain, 4X; **B -** Verhoeff's Elastic stain 4X; **C -** Verhoeff's Elastic stain, 10X).

**Figure 3:**
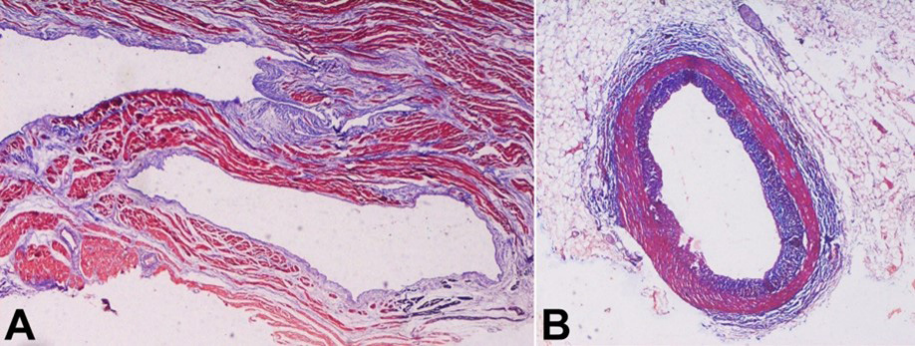
Photomicrographs of the coronary arteries. **A -** shows the left circumflex artery with a thin vessel wall surrounded by the cardiac muscle (Masson trichrome stain, 20X); B**-** histologic cross-section of coronary artery showing normal tunica intima, the thickness of tunica media, and tunica adventitia of the right coronary artery (Masson trichrome stain).

The histopathological examination revealed the left anterior descending and circumflex arteries’ walls were thinned out, while the right coronary artery was normal. The muscular layer was missing and underdeveloped in the left anterior descending (Figure 22B and 2C) and left circumflex arteries (Figure 3A). The tunica media or muscle layer was thin and absent at places along the vessels’ wall, evidenced by the Masson trichrome staining and Verhoeffs stain in left anterior descending artery (Figure 2A, 2B and 2C).The right coronary artery showed normal arterial wall histology (Figure 3B).

**Figure 4:**
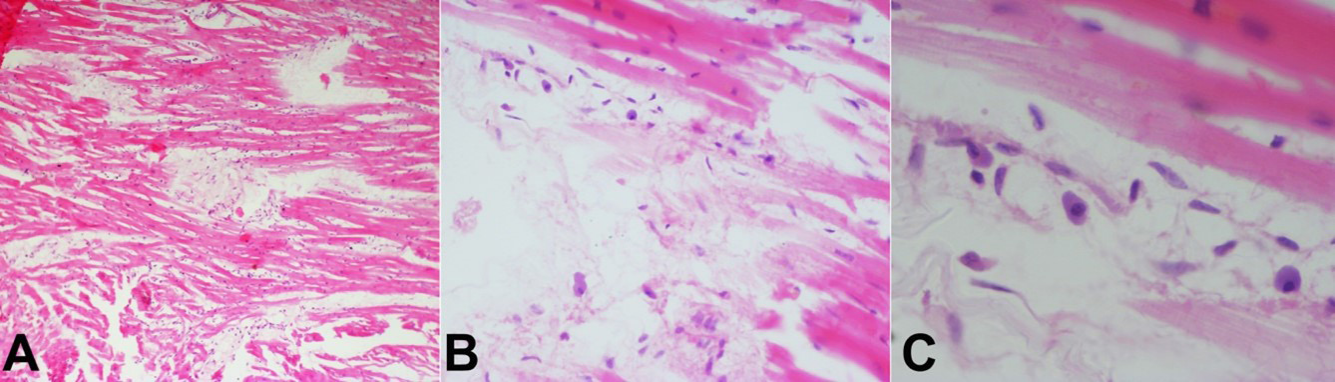
Photomicrographs of myocardial muscle: **A -** shows the separation of myocardial fibers and edema (H&E, 4X); **B** and **C -** show inflammatory cells comprising of macrophages and lymphocytes in between myocardial fibers (H&E, B-40X, C-100X).

The myocardium examination showed evidence of recent myocardial infarction represented by myocardiocytes interspersed by edema (Figure 4A), and early neutrophilic infiltration (Figure 4B) with reactive nuclei of myocardium (Figure 4C). The remaining organs showed only congestion on HPE.

## DISCUSSION

The significant reduction in the luminal diameter and length of one or more main coronary arteries characterizes hypoplastic coronary artery disease (HCAD).^14,15^ According to the Indian population study on the normal diameters of the left main coronary artery, the proximal left anterior descending artery, proximal right coronary artery, and proximal left circumflex artery are respectively 4.08±0.44mm, 3.27±0.23mm, 3.20± 0.37mm, 2.97±0.37 mm.^16^ Most of HCAD cases are more frequently detected during autopsies than by *in vivo* coronary angiography. HCAD was initially reported in 1970 by Ogden.^17^ A small coronary artery or arteries, the absence of compensatory collateral circulation, microscopic evidence of myocardial ischemia, and the exclusion of other causes of death have all been considered the cause of death due to coronary artery hypoplasia.^18^Cases of HCAD with unexplained episodes of syncope in young have been reported. Other symptoms include palpitation, exertional dyspnea, and chest pain.^8,18,19^

The case presented herein has a history of exertional dyspnea and syncope, and all the coronaries’ lumens’ diameters were narrowed even lesser than the recommended diameter of 1.5 mm reported in the literature.^8,10,11^ Giorgio et al.^18^ reported two autopsy cases of HCAD in an adult and a child. The first was a sudden death case in a 35-year-old female with a prior history of dyspnea on exertion. On autopsy, the left circumflex coronary artery was markedly hypoplastic, measuring less than 1.0 mm in diameter, with a scar of old myocardial infarction and changes of recent myocardial infarction. In the second case, a 9-year-old girl died during a physical activity at school, whose circumflex and anterior descending branches measured 0.8 mm in diameter (normal: 1.8±0.5 mm for age)^20^ with microscopic findings of ischemia. Ischemic changes are frequent findings seen in HCAD.^4,18,21^ Riede et al.^4^ reported hemorrhagic infarction in a case of HCAD. In another case, a 45-year-old woman who had collapsed and passed away had a dominating, small-caliber right coronary artery, which supplied the posterior interventricular septum with acute and chronic ischemic alterations.^5^ Histopathological examination of the heart in HCAD had shown ischemic changes in myocardial tissue with a decreased luminal diameter of coronaries. However, there is a lack of study and information on the microscopic architecture of the involved vessel tissue. Although microscopic findings of myocardium and coronaries were previously described,^4,5^ the details of the muscular layer of the coronary artery are lacking. Arteries have more smooth muscle and elastic tissue than veins.^22^ With such a relatively thick wall, arteries can also retain the patency during the diastole and systole during exertion. In our case, there was a missing and undeveloped muscular layer or the tunica media of the left anterior descending and left circumflex artery, which is a deviation from the normal histology of the coronary artery and resembles the microscopic architecture of a vein. This anomaly must have led to the collapse of vessels, making them unable to maintain patency during exertion leading to death due to myocardial infarction.

According to recent advanced studies on cases with HCAD, both congenital and acquired factors influence coronary artery flow.^14,15^ The formation of the coronary arteries has been the subject of some research. It has been suggested that some genetic variables may be connected to HCAD. In a case of a 10-year-old boy who presented with chest pain and syncope following exertion, the diagnosis of HCAD and abnormal origin of the coronary was made through radio imaging. A new mutation site was identified in the *NOTCH1* gene, which encodes Neurogenic locus notch homolog protein 1.^23^ In HCAD cases with aborted sudden cardiac death, patients have survived with no symptoms with proper follow-up and medical care.^8,10^ Implantable cardioverter-defibrillators are useful for aborting sudden cardiac death as secondary prevention.^7,14,15,24^ Patients with HCAD who develop ischemic cardiomyopathy and end-stage cardiac failure may need a heart transplant.^21^ Although HCAD is an entity with potential *in vivo* diagnosis, most cases are unveiled during autopsy. HCAD is very uncommon, and indications of coronary anomalies should be sought during autopsies in cases of unexpected sudden deaths, particularly in younger age groups.

## CONCLUSION

Coronary anomalies should always be sought during autopsies of unexpected sudden deaths. The microscopic characteristics of the myocardium were previously described; however, the characteristics of the muscular layer of coronaries was not covered. Studies and case reports described only a narrow luminal diameter or shorter course of coronary arteries in HCAD. Our case reports the detailed microscopic analysis of coronary arteries and shows the deficiency in tunica media visualization involving arteries in HCAD.
